# Bioinspired nanocoatings for biofouling prevention by photocatalytic redox reactions

**DOI:** 10.1038/s41598-017-03636-6

**Published:** 2017-06-15

**Authors:** Priyanka Sathe, Karthik Laxman, Myo Tay Zar Myint, Sergey Dobretsov, Jutta Richter, Joydeep Dutta

**Affiliations:** 1 Department of Marine Science & Fisheries, College of Agricultural & Marine Sciences, Sultan Qaboos University, P.O. Box 34, Al Khoud, 123 Sultanate of Oman; 2 Chair in Nanotechnology, Water Research Center, Sultan Qaboos University, P.O. Box 17, Al Khoud, 123 Sultanate of Oman; 3 Functional Materials Division, Department of Applied Physics, School of Engineering Sciences, KTH Royal Institute of Technology, Isafjordsgatan 22, SE-164 40, Kista Stockholm, Sweden; 4 Department of Physics, College of Science, Sultan Qaboos University, PO Box 36, Al Khoudh, Muscat 123 Sultanate of Oman; 5 Center of Excellence in Marine Biotechnology, Sultan Qaboos University, P.O. Box, 50 Al Khoud, 123 Sultanate of Oman; 6 Institute for Chemistry and Biology of the Marine Environment (ICBM), University of Oldenburg, Ammerländer Heerstraße 114, 26129, Oldenburg, Germany

## Abstract

Aquaculture is a billion dollar industry and biofouling of aquaculture installations has heavy economic penalties. The natural antifouling (AF) defence mechanism of some seaweed that inhibits biofouling by production of reactive oxygen species (ROS) inspired us to mimic this process by fabricating ZnO photocatalytic nanocoating. AF activity of fishing nets modified with ZnO nanocoating was compared with uncoated nets (control) and nets painted with copper-based AF paint. One month experiment in tropical waters showed that nanocoatings reduce abundances of microfouling organisms by 3-fold compared to the control and had higher antifouling performance over AF paint. Metagenomic analysis of prokaryotic and eukaryotic fouling organisms using next generation sequencing platform proved that nanocoatings compared to AF paint were not selectively enriching communities with the resistant and pathogenic species. The proposed bio-inspired nanocoating is an important contribution towards environmentally friendly AF technologies for aquaculture.

## Introduction

Biofouling in aquaculture industry is a complex and recurrent global problem^1^. A typical aquaculture infrastructure comprises of a variety of immersed components and structures, such as nets, connecting ropes, buoys and cages, which form an integral part of breeding environments^2^. Structures immersed in seawater serve as ideal sites for the development of biofouling, which refers to the undesirable growth of micro- and macro-organisms on them^3^. Biofouling can lead to cage deformation, blockage of water exchange across nettings, degradation of water quality etc., all of which influence the health and, thus, growth of the fish^2^. Biofouling can thus have adverse effects on culture species as well as aquaculture infrastructures^4^. Methods to control biofouling form a significant part of the cost accounting, with average estimations being 5–10% of the total production costs^4^.

Commonly used biofouling prevention techniques involve the use of copper based antifouling (AF) paints applied to the nets, combined with frequent washing to prevent biomass accumulation^5^. However, the decay of the biocidal effects with time along with constant leaching of heavy metal ions from the paints poses a significant threat to the marine environment^1, 6, 7^. Several reports also suggest that upon long term utilization, fouling organisms can develop resistance to copper based antifouling paints^8^. Thus, there is a significant demand for developing an alternative “green antifouling” technology for application in the aquaculture industry.

Environmentally safe and long lasting solutions can be developed by mimicking some of the naturally evolved and effective antifouling techniques used by aquatic organisms^9^. These approaches usually include production of chemical molecules along with specific physical features on the organism’s surface (like micro- topography, wettability etc.) to prevent the settlement of fouling organisms^10–12^. Several reports have focused on developing bio-inspired eco-friendly AF surfaces^13, 14^. Certain algal species defend themselves from biofouling by producing reactive oxygen species (ROS) such as hydroxyl radicals and peroxides^15–17^. This mechanism is in fact similar to the working of a photocatalytic material^18^, which led us to investigate the antifouling capabilities of nanostructured semiconductor oxides with considerable success (under visible light excitation) in both a laboratory and mesocosm settings^18, 19^. However, the commercial viability of the nanostructured coatings can only be established by testing their antifouling activity in field conditions and under naturally occurring conditions over a reasonable period of time.

This study elaborates on the practicality of the technique by testing the biofouling resistance of nanostructural modification of fishing net immersed under static conditions in a tropical marine environment (Sea of Oman) for a period of one month. Surface modification of the net was carried out by growing nanorods of a photocatalytic material, zinc oxide (ZnO). The antifouling performance of the ZnO nanocoatings were investigated by detailed characterization of abundance and diversity of prokaryotic and eukaryotic organisms on the netting surfaces. High throughput sequencing (Illumina MiSeq) was also used to identify and compare the species of biofouling organisms accumulated on the nets. The performance of the nanostructured nets was evaluated and compared to the results obtained using commercial copper based antifouling paint, wherein it was observed to perform significantly better over the test duration.

## Results

### Structural and chemical characterization of coatings

**Figure 1:**
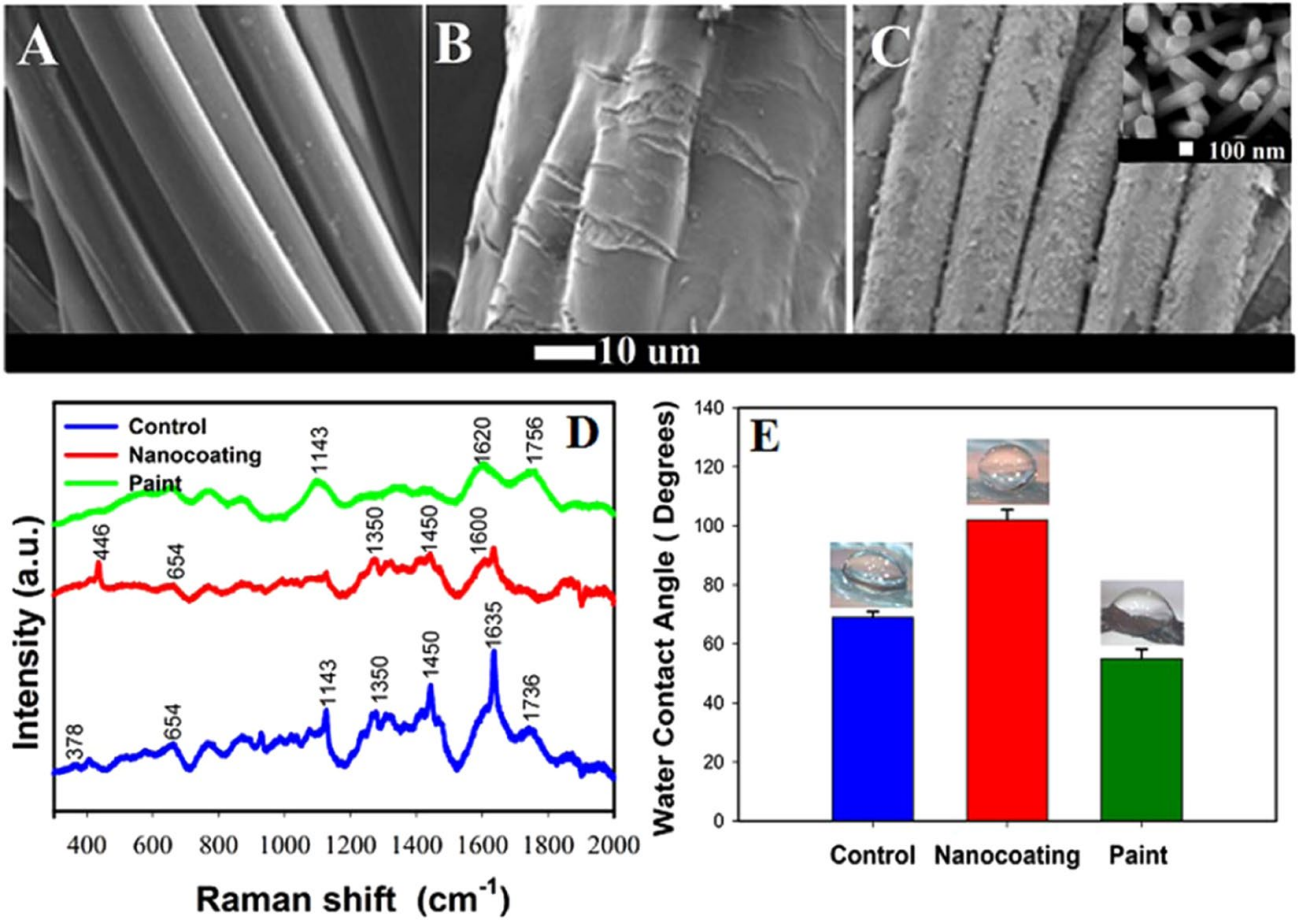
Morphology, wetting and Raman spectroscopy of coatings. Scanning electron micrographs of (**A**) Single fibre of control nylon 6 net. (**B**) Single fibre of nylon 6 net after coating with AF paint. (**C**) Single fibre of nylon 6 net after coating with ZnO nanorods. (**D**) Raman spectra for control, AF painted and nanocoated nylon 6 net substrate. (**E**) Water contact angle measurement for control, AF painted and nanocoated net substrates.

Figure 1 shows the scanning electron micrographs (SEM) of a single fibre of plain nylon net (Fig. 1A), fibre coated with commercial antifouling paint (Fig. 1B) and fibre with well distributed ZnO nanorods having an average length and width of 1.5 ± 0.4 µm and 100 ± 7 nm, respectively (Fig. 1C). The ZnO nanorods were observed to grow almost perpendicular to the net surface, essentially enabling increased rod density. Raman spectroscopy illustrated the prominent Nylon 6 features of unmodified net. However these features were diminished for the painted net, suggesting a uniform thick coating, while for the ZnO nanorod coated net they were present alongside specific zinc oxide peaks, suggesting intermittent surface coverage (Fig. 1D). Raman spectra of the nanorods coated nets exhibited five prominent peaks at 446, 654, 1350, 1450 and 1600 cm^−1^ in addition to the contribution from nylon 6 (Fig. 1D).

Further characterization of the samples (Supplementary Fig. 3) under X-ray photoelectron spectroscopy (XPS) showed the presence of oxygen (Binding energy, BE of 530.1 eV) and adventitious carbon (BE of 284.6 eV) in the all net samples; with additional peaks of Zn 3p (88.6 eV), Zn 3 s (139.1 eV) and Zn 2p (1021.7 and 1044.6 eV) for the nanorod coated nets and Cu 2p (961 eV) and Zn 2p (1021.7 and 1044.6 eV) for the painted nets, which is in agreement with the manufacturers specification of Hempanet paint^20^. Total surface area using BET indicated that the nanorod coated nets showed highest surface area compared to control and painted samples, which is attributed to the additional surface area available from the thick nanorod growth (Supplementary Table 2). Water contact angle measurements of the samples were 69 ± 2° for the control sample, that reduced to 55 ± 3° upon painting the net with a commercial paint, but improved dramatically to 102 ± 3° for the nanorod coated net (Fig. 1E).

**Figure 2:**
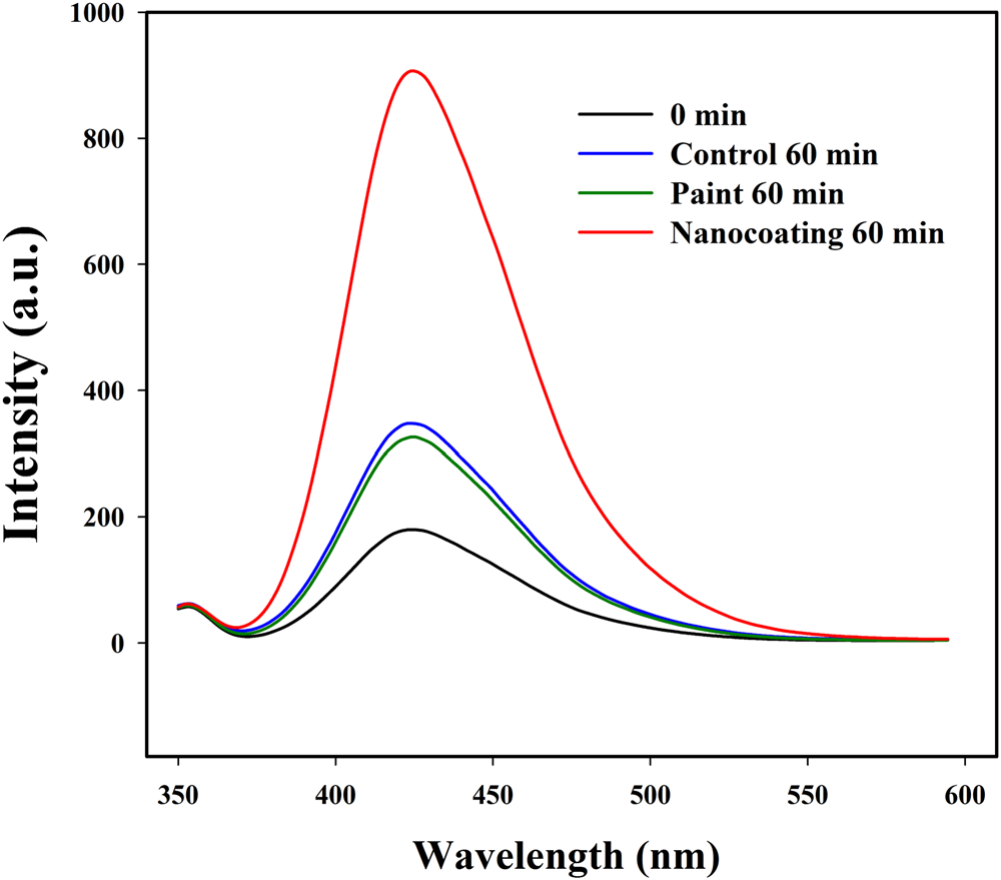
Photoluminescence spectra of Terapthalic acid after treatment with coatings. Photoluminescence spectra of Terapthalic acid treated with control, nanocoated and painted net substrate illuminated by solar stimulator for 1 hour.

In order to investigate the production of ROS from nanocoatings upon light activation, a separate photocatalysis experiment was performed under artificial sunlight. Terephthalic acid (THA) was used as an indicator, which combines with hydroxyl radicals (**·**OH) to form a florescent product quantified by using photoluminescence spectroscopy^21^. Figure 2 shows the production of hydroxyl radicals in the presence of nanocoatings, painted surface and control nets during photocatalysis. Significant production of hydroxyl radicals could be observed in the presence of nanocoated substrates under artificial sunlight (ANOVA, Dunnett, df = 6, p = 0.000001). Whereas painted substrate did not show significant production of hydroxyl radicals (ANOVA, Dunnett, df = 6, p = 0.24).

### Prevention of biofouling

**Figure 3:**
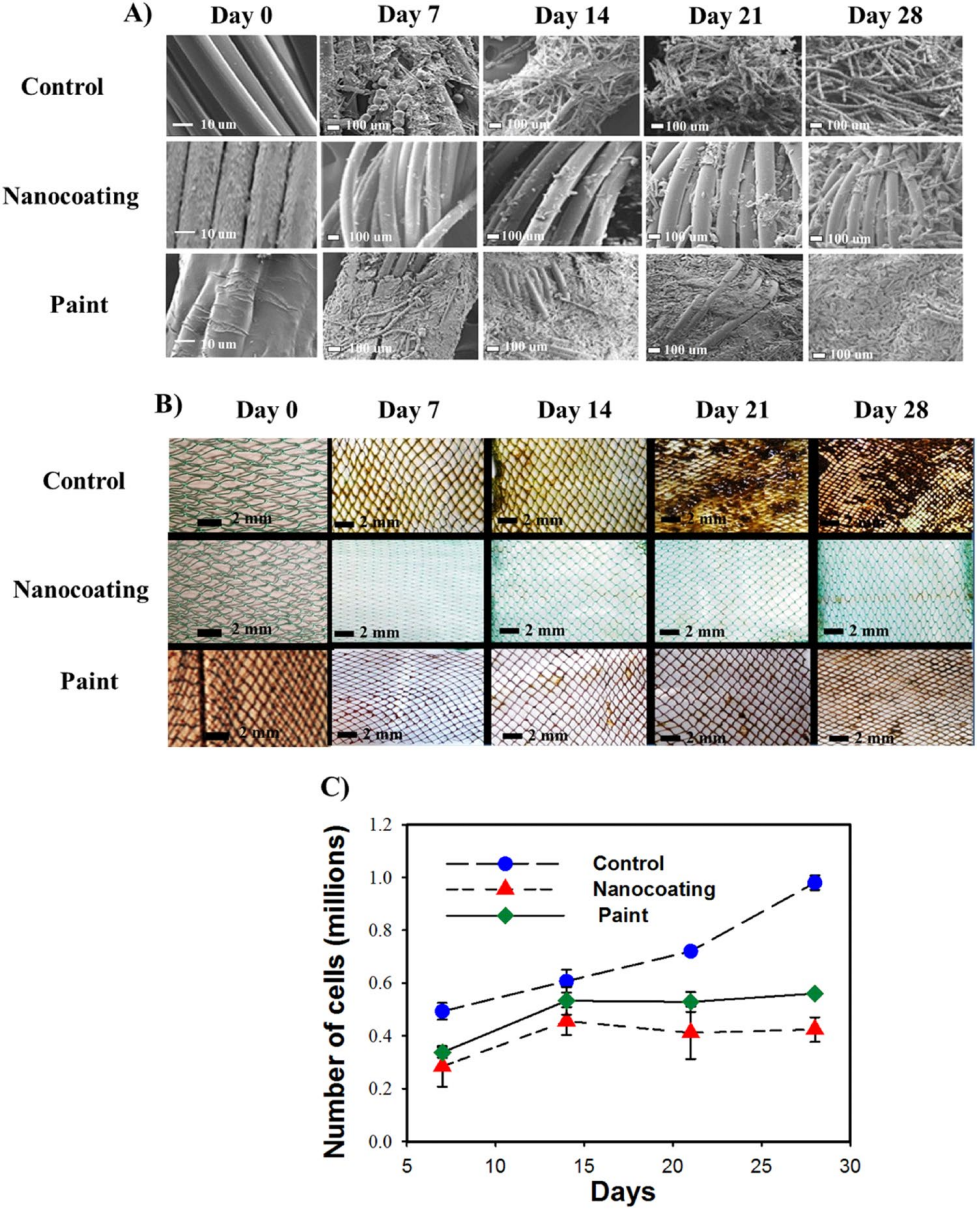
Surface coverage and total microbial abundance of the coated substrates. (**A**) Scanning electron micrographs of control, nanocoated and AF painted net substrates over the period of 4 weeks. (**B**) Sample optical micrographs of control, nanocoated and AF painted substrates over period of 4 weeks. (**C**) Variation in the total number of bacterial cells on the control, nanocoated and AF painted net substrates per millilitre as measured by flow cytometry over the period of 4 weeks. Day 0 counts are not shown as all the counts were zero. Reported values show average readings of 3 replicates ± standard deviation.

Representative scanning electron micrographs (SEM) showed clearly different attachment of fouling microorganisms to the control, painted and nanocoated netting substrates (Fig. 3A). Optical micrographs and scanning electron micrographs revealed that lowest biofouling coverage could be observed on the nanocoated substrates compared to control or the biocidal paint modified netting over the period of one month (Fig. 3B). Factorial ANOVA analysis showed that treatment (ANOVA, F = 421.855, df = 2, p = 0.0000001) and the experimental duration (ANOVA, F = 184.829, df = 3, p = 0.0000001) affected the density of fouling microbes significantly either independently and/or in combination (ANOVA, F = 37.691, df = 6, p = 0.0000001). Nanocoatings (3- fold reduction in abundance) and paint (2- fold reduction in abundance) inhibited fouling microorganisms over the test period which was evident from the low microbial abundance compared to the un-coated control substrates (Fig. 3C). Microbial abundance on the nanostructured surface was also found to be significantly lower (ANOVA, HSD, df = 24, p = 0.0000129) compared to the control at all four sampling times (Fig. 3C).

### Metagenomic analysis of communities

**Figure 4:**
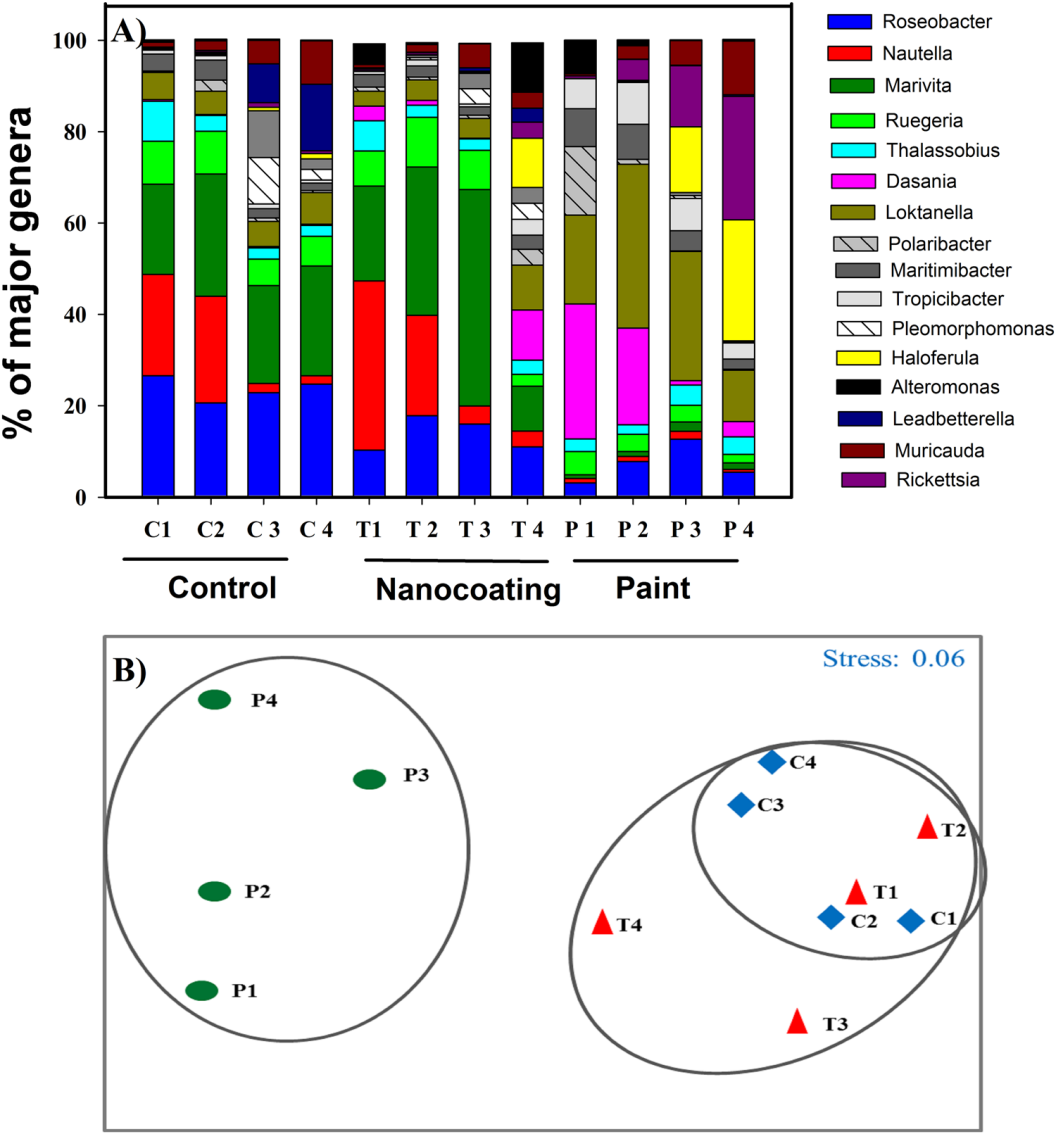
Community composition of major fouling bacteria and cluster analysis. (**A**) Stacked bar chart showing the relative abundance (%) of major bacterial genera present on the control (**C**), nanocoated (T) and AF painted (P) net substrates during exposure in marina. 1–4 represents samples collected on weeks 1 to 4. Reported values represent average value obtained from three replicates. Bacterial genera contributing <5% are not shown. (**B**) MDS plot showing the clustering of major bacterial genera present on the control (**C**), nanocoated (T) and AF painted (P) net substrates during one month exposure in marina. 1–4 represents samples collected on weeks 1 to 4.

To further explore reasons of the better antifouling efficiency of nanocoatings over antifouling paint, metagenomic analysis was performed. We believe this is the first time the compositions of marine biofilms were studied using MiSeq Illumina next generation sequencing of 16S and 18S rRNA genes. Among prokaryotes, phylum *Proteobacteria* dominated the biofilms on all samples. Control and nanocoated substrates consistently showed higher percentage of the bacterial class *Alphaproteobacteria*, whereas painted substrate showed *Gammaproteobacteria* and *Flavobacteria* (Supplementary Fig. 5). The proportions of *Alphaproteobacteria* were found to be lower on the painted samples during the period of investigations. In our study, bacteria belonging to genera *Roseobacter* and *Marivita* were found to be the most dominant in the samples collected from the control and nanocoated nettings (Fig. 4A). On the other hand, biofilms on paint was dominated by genera *Dasnia*, *Haloferula* and *Rickettsia* (Fig. 4A). Cluster analysis showed presence of unique clusters in the samples collected from the painted net substrate, whereas control and nanocoated substrates were observed to possess similar composition shown by overlapping clusters. (Figure 4B). Cluster analysis was in good agreement with the diversity analysis, which indicated that there was no significant difference between community compositions on control or nanocoated substrates.

**Figure 5:**
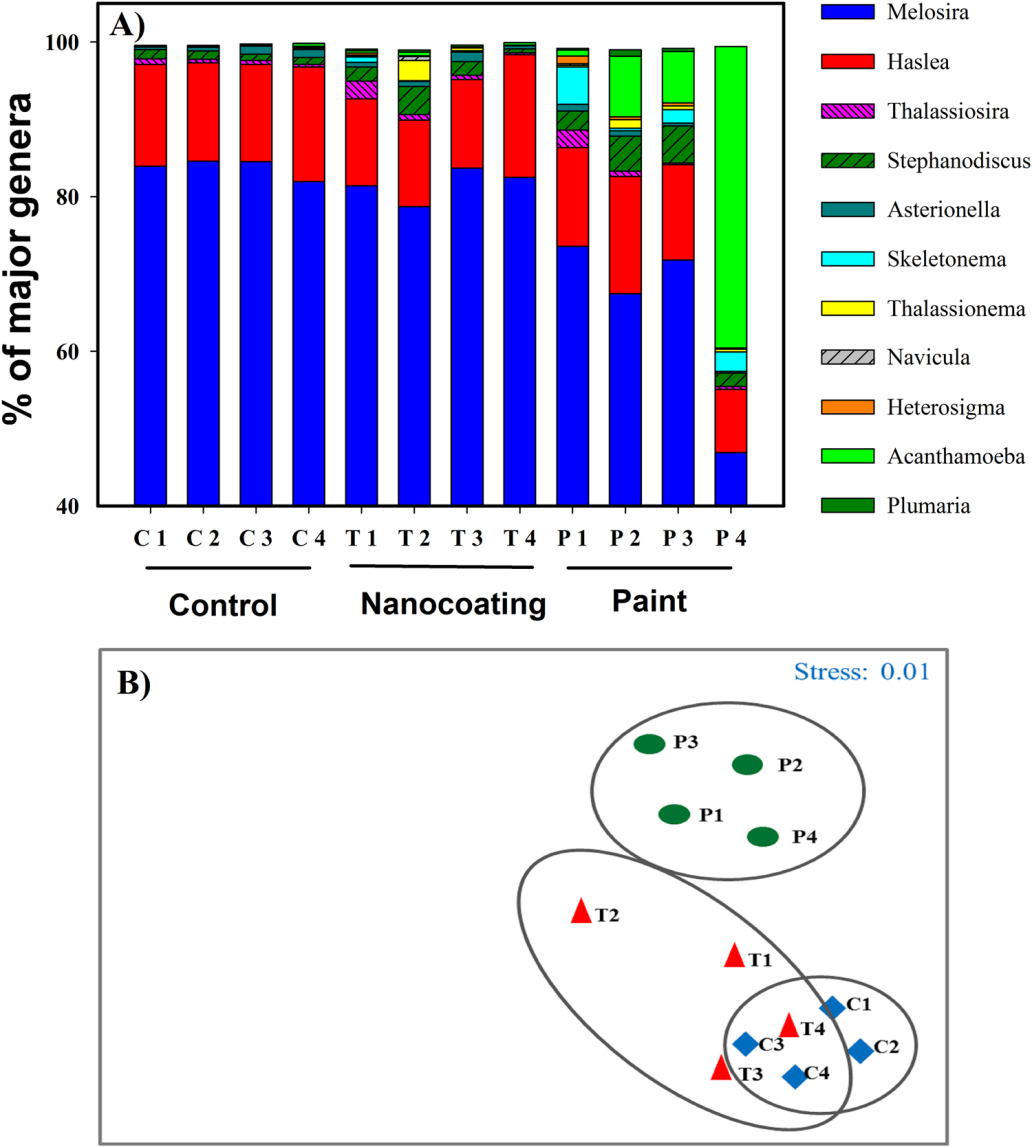
Community compositions of major fouling eukaryotes and cluster analysis. (**A**) Stacked bar chart showing the relative abundance (%) of major eukaryotic genera present on the control (**C**), nanocoated (T) and AF painted (P) net substrates during exposure in marina. 1–4 represents samples collected on weeks 1 to 4. Reported values represent average value obtained from three replicates. Eukaryotic genera contributing <1% are not shown. B) MDS plot showing the clustering of major eukaryotic genera present on the control (**C**), nanocoated (T) and AF painted (P) net substrates during one month exposure in marina. 1–4 represents samples collected on weeks 1 to 4.

Among eukaryotes, *Bacillariophyta* and *Protozoa* dominated the biofilms in all samples (Fig. 5A). Control and nanocoated substrates consistently showed higher percentages of the diatom genera *Melosira* and *Haslea*, whereas on the painted surface, presence of higher percentages of the protozoa *Acanthamoeba* along with the diatom *Melosira* could be observed. Eukaryotic protozoan belonging to genus *Acanthameoba* were exclusively found on the painted surfaces (Fig. 5A). However the percentage of *Acanthamoeba* was found to gradually increase with the progression of experiment over the month, with simultaneous gradual decrease of the presence of diatoms. A dominance of the diatoms *Haslea* sp. and *Acanthamoeba* sp. at the end of 4^th^ week could be observed on the AF painted substrates. Similarly to prokaryotes, cluster analysis showed presence of unique cluster only for the painted net substrates (Fig. 5B) whereas control and nanocoated substrates showed overlapping clusters. This is in good agreement with observations made during the diversity analysis, indicating that there was no significant difference between community compositions on control and nanocoated substrates.

SIMPER analysis of bacterial communities revealed that total dissimilarity of bacterial communities developed on control and paint surfaces was the highest at about 39.67% followed by dissimilarity of bacterial communities developed on nanocoated and painted surfaces which was about 36.80% and the least dissimilarity was observed between control and nanocoated surface at about 22.69%, respectively (Table 1). Bacteria belonging each genera *Nautella*, *Marivita* and *Leadbetterella* accounted for more than 10% of dissimilarity each, between communities developed on the control and nanocoated substrates (Table 1). *Marivita*, *Dasnia* and *Nautella* genera accounted for more than 8% of dissimilarity each, between communities developed on the control and painted surfaces. The same genera contributed to the dissimilarity between communities developed on nanocoated and painted substrates (Table 1). On a similar note, SIMPER analysis for eukaryotic communities revealed that highest dissimilarity is observed between control and painted surfaces at 23.98%, followed by nanocoated and painted surfaces at 22.30% and the least between control and nanocoated surfaces at 9.05% (Table 2). Eukaryotes belonging to each genus *Thalassiosira*, *Stephanodiscus* and *Thalassionema* accounted for more than 10% of dissimilarity each, between communities developed on the control and nanocoated substrates (Table 2). *Acanthamoeba*, *Skeletonema, Melosira* genera accounted for more than 10% of dissimilarity each, between communities developed on the control and paint. The same genera contributed to the dissimilarity between communities developed on nanocoated and painted substrates (Table 2). Our results also showed that the highest number of unique operational taxonomic units were observed in the biofilms formed on the painted surfaces (week 3; P3, OTU = 895), while the lowest number of OTUs were observed in samples obtained from the nanocoated surfaces (week 4; T4, OTU = 225) (Supplementary Table 1). Generally, more OTU’s were detected in the samples obtained from control and painted nettings compared to nanocoated nettings demonstrating that overall diversity of microorganisms is lower on the nanorod coated net surface.

**Table 1:** SIMPER analysis for estimation of bacterial genera contribution to total dissimilarity.

Genera	Control vs. Nanocoating (22.69%)	Control vs. Paint (39.67%)	Paint vs. Nanocoating (36.80%)
Contribution (%)			
*Marivita*	10.30	**12.55**	**13.60**
*Dasania*	6.73	8.89	7.67
*Roseobacter*	6.04	7.77	—
*Loktanella*	—	7.39	8.48
*Rickettsia*	—	7.33	7.61
*Nautella*	**13.17**	6.97	10.47
*Haloferula*	5.29	6.93	7.50
*Leadbetterella*	9.19	5.42	—
*Tropicibacter*	—	5.18	—
*Pleomorphomonas*	6.59	—	—
*Alteromonas*	6.54	—	—
*Muricauda*	5.23	—	

The contribution of particular genus of bacteria towards the total dissimilarity (%) between the bacterial communities using SIMPER analysis.

**Table 2:** SIMPER analysis for estimation of Eukaryotes genera contribution to total dissimilarity.

Genera	Control vs. Nanocoating (9.05%)	Control vs. Paint (23.98%)	Paint vs. Nanocoating (22.30%)
Contribution (%)			
*Acanthamoeba*	10.92	**25.57**	**24.02**
*Skeletonema*	7.74	15.73	15.29
*Melosira*	—	15.56	15.28
*Thalassionema*	11.84	8.30	8.21
*Thalassiosira*	**16.55**	7.18	8.11
*Haslea*	9.20	7.03	8.11
*Stephanodiscus*	12.08	6.96	6.83
*Plumaria*	7.94	—	—
*Asterionella*	6.65	—	—
*Navicula*	6.36	—	—
*Heterosigma*	5.97	—	—

The contribution of particular genera of eukaryotes towards the total dissimilarity (%) between the eukaryotic communities using SIMPER analysis.

## Discussion

Typically, hydrophobic low energy surfaces with initial surface tensions ranging between 20 and 30 mN/m have low adhesion towards fouling organisms^22^. It is well known that application of AF paint renders substrates hydrophilic^23^, while application of zinc oxide nanorods on nettings lead to relatively hydrophobic surfaces following similar observations made elsewhere^24, 25^. Generally, ZnO nanocoatings exhibit hydrophilic properties due to their inherent properties^24^. However, when grown as micro or nanopillars, the reduced contact area of the NR’s with water leads to the generation of a low energy surface, whose hydrophobicity can be tuned by controlling the density, height, diameter or surface energy of the rods^25, 26^.

**Figure 6:**
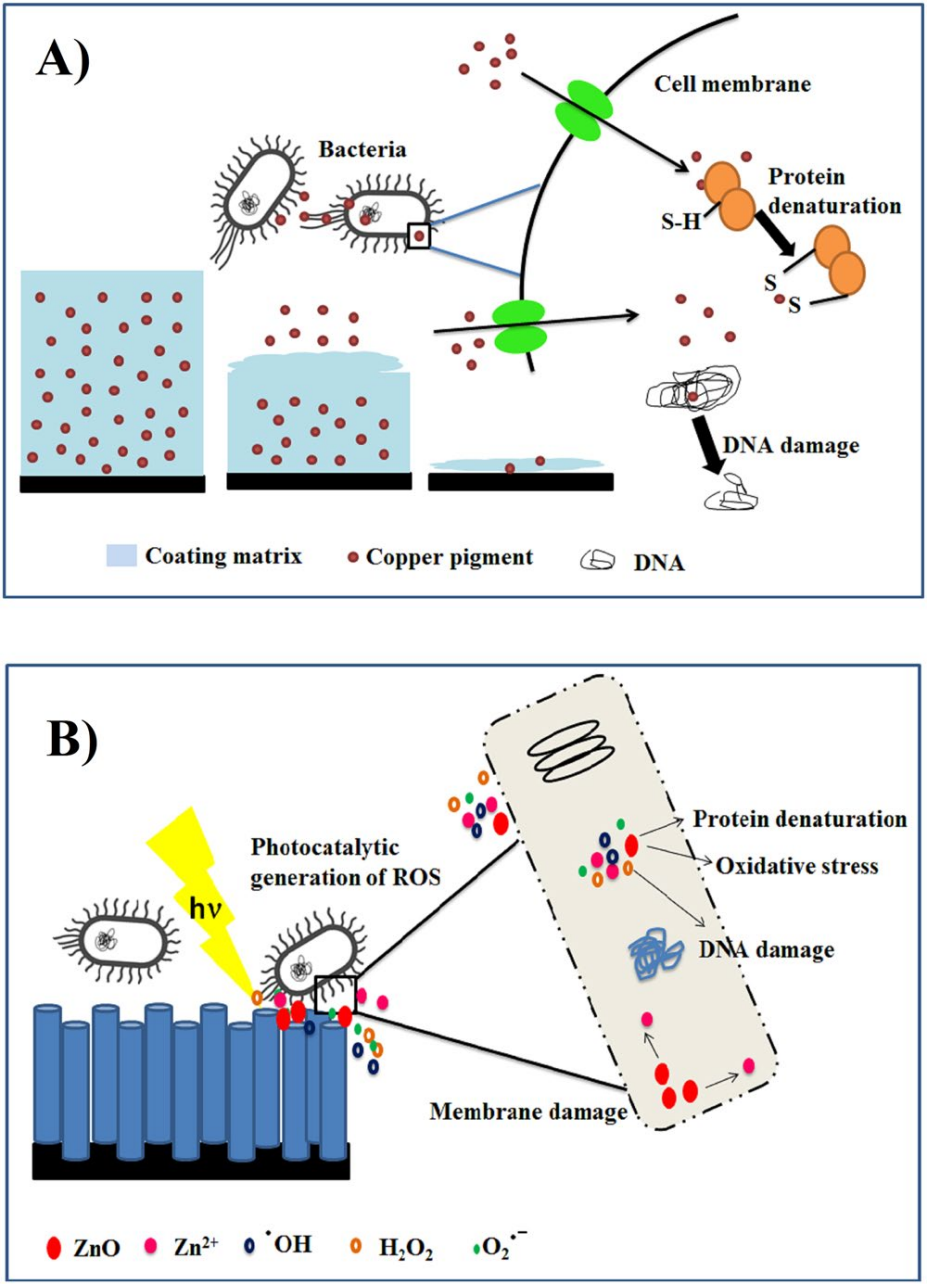
Schematic representation for mechanism of action. Schematic representation for mechanism of antifouling action for (**A**) copper based antifouling paint (**B**) for zinc oxide nanorod coated substrates.

The antifouling efficiency of ZnO nanocoatings and paint was analysed using metagenomic approaches, which are fast gaining popularity in the studies of marine ecology of environmental biofilm assemblage^27, 28^. By taking advantage of the faster and more detailed genetic profiling of complex environmental samples from MiSeq Illumina next generation sequencing of 16S and 18S rRNA technique^29–32^, a comprehensive database of the microbial diversity was obtained (see results section). This yields an accurate picture of the dependence of fouling communities and the antifouling mechanisms of the painted and ZnO nanorod coated substrates. Copper based antifouling paints prevent biofouling by the release of biocidal material at a rate high enough to maintain toxicity in the surrounding environment. The toxic layer hinders metabolic activities of fouling bacteria, larvae of invertebrate’s and algal spores, thus preventing their settlement on the AF painted surfaces^33^. Typically the biocidal material comprising of copper and zinc ions leaches out from the cuprous oxide and zinc oxide (binder) matrix of the paint (Fig 6A). Both ions in high concentrations are toxic to all microorganisms as they can displace/substitute the essential metals from their native binding sites due to their greater affinity towards thiol- containing groups and oxygen sites (Fig 6A)^34^. Toxicity can also occur due to alterations in the conformational structure of nucleic acids, proteins and adverse effects of copper on cellular oxidative phosphorylation and osmotic balance^35^. However, both these mechanisms have a major drawback as certain bacteria can build a resistance to copper, thus leading to selective growth and colonization as is observed from the results obtained on copper-based coatings^36, 37^.

Alternatively, ZnO nanocoating prevents biofouling by producing reactive oxygen species under visible light irradiation, a process known as photocatalysis. Visible light photocatalysis is an environmentally friendly technique requiring a photocatalytic material like ZnO to degrade a wide variety of contaminants^19, 38–40^. Upon irradiation, a photocatalytic material generates a multitude of electron-hole pairs, which in an aquatic environment, leads to the generation of reactive oxygen species (ROS) like hydroxyl radicals (•OH), hydrogen peroxide (H_2_O_2_) and superoxide ions (O_2_^-^)^40^. ROS are known to cause microbial cell membrane damage and increase oxidative stress ultimately leading to cell death (Fig. 6B)^41, 42^. One of the well-known targets of ROS is microbial DNA^43^, where processes like base oxidation (chiefly guanine) and strand breaks lead to cell death^44^. Additionally, zinc ions which leach from the nanocoatings are known to bind with pili of the bacteria and affect the bacterial growth cycle^45^. This process is in fact important to protect the net surface during the absence of light, which was close to 10 hours a day. However it is important to note that unlike the AF paint which has a high ion release rate affecting target as well as non-target organisms in the vicinity, zinc ions are released very slowly (~20 ppm per year under constant illumination at peak sunlight intensity), as sea water is a natural buffer and has a pH (~8.0) where ZnO is extremely stable^46^. Also the surface radical species generated upon photo absorption are short lived and are confined in extremely localized zones, which while being non-selective, but mimicking the natural defence mechanism used by several marine microalgae, neutralizing those microorganisms only at the proximity of the nanocoated surfaces^45^. This combined with the fact that zinc is an essential constituent of DNA and RNA polymerase enzymes and that 15–30 mg/kg of zinc is considered essential for ideal fish growth in the aquaculture industry, makes the process an imitation of naturally occurring mechanisms and an environmentally friendly method to prevent biofouling^47^.

Further evidence on ROS generation is obtained from the diversity analysis, where it can be observed that communities formed on nanocoatings were similar to ones formed on control substrates, both of which were different from the AF painted substrates. Previous reports have suggested that *Alphaproteobacteria* dominates marine biofilms developed on various types of immersed artificial substrates^30, 32, 48^. Commercial antifouling paints have been reported to be dominated by bacteria belonging to classes *Alphaproteobacteria*, *Gammaproteobacteria, Firmicutes* and *Bacteroidetes* ^49^. Our results indicate that dominant accumulation of bacteria belonging to classes *Alphaproteobacteria*, *Gammaproteobacteria*, *Firmicutes* and *Bacteroidetes* occur on the AF painted nets, while the natural selection favours genus *Roseobacter* on the nanorod coated and control nets (absent in AF painted nets). *Alphaproteobacteria* and *Gammaproteobacteria* are Gram negative bacteria belonging to phylum *Proteobacteria*, wherein *Alphaproteobacteria* comprises of various phototrophs and other symbiotic bacteria and *Gammaproteobacteria* consists of ecologically and medically important bacterial strains^50^. *Firmicutes* represents bacterial phylum containing Gram positive bacteria that are known to produce endospores and thrive in extreme environments^50^. *Bacteroidetes* on the other hand represent bacterial phylum containing Gram negative bacteria which are abundantly present in seawater and sediments^50^. Some strains of bacteria from the *Roseobacter* clade have been used in aquaculture as probiotic strains, as they can kill fish pathogens such as *Vibrio anguillarum*, through tropodithietic acid production^51^. They have been utilized as biofilters and help in reducing the mortality of fish larvae^52^. Additionally the diatom *Haslea ostraria* (abundant in the biofilm samples collected from nanorod coated and control nets) is a natural antibiotic producer which was reported to actively reduce algal blooms^53^. Additionally, the AF coated nets were found to harbour bacteria like *Dasnia*, *Haloferula* and *Rickettsia* which are well known human and fish pathogens, and are known to clog biofilters in aquaculture installations^54^. The painted nets were also found to harbour eukaryotic protozoans belonging to the genus *Acanthameoba*, which are disease causing protozoans abundantly found in seawater, sewage water etc. and are known to interact with intracellular pathogenic bacteria^55^, increasing their survival rate, virulence and biocidal resistance^56, 57^.

One of the probable reasons for the selective settlement of species could be due to the high number of copper resistant bacteria present on copper painted surfaces which attracts protozoan predators^55^. Thus AF painted nets provide a selective environment for harmful, pathogenic prokaryotic and eukaryotic organisms which pose a significant threat to aquaculture and health, while ZnO nanocoated and control nets do not harbour resistant strains, while providing an environment where microorganisms beneficial to the industry and health are dominant. Additionally the month long experiments showed that the nanocoated nets accumulated 3-fold lower densities of fouling microorganisms compared to the control and were more effective in the prevention of microbial biofouling compared to AF painted nets over temperature ranges between 20–37 °C (Supplementary Fig. 6).

In summary, we have successfully developed sunlight-responsive antifouling ZnO nanorods coated fishing nets that are more efficient at mitigating biofouling compared to commercial biocidal paints. The prevention mechanism is an imitation of naturally occurring processes, leading to selective accumulation of microorganisms beneficial to the aquaculture industry and human health. The proposed scalable and low-cost approach with real time applicability is an important contribution towards the effective development of solar-assisted, environmentally friendly antifouling technologies targeted for the aquaculture industry.

## Methods

### Materials

Fishing monofilament nylon net was purchased from Rayee International Corporation (China) and was used as a support substrate for the experiment. Commercial biocidal paint Hempanet, 7177A was obtained from Hempel A/S (Denmark). Sodium hydroxide and zinc acetate were supplied by MERCK (Germany) while Dodecanethiol, zinc nitrate hexahydrate, hexamethylenetetramine and terephthalic acid were from supplied from Sigma Aldrich (USA). SYBR Green I flurochrome was obtained from Molecular Probes, Invitrogen (USA), Power Biofilm MoBio Kit from MoBio (USA) and the cell strainers were obtained from Falcon^TM^, Fischer Scientific (USA).

### Surface modification

Fishing monofilament nylon net was used as a support substrate for growing zinc oxide nanorods (nanocoating) and also for coating with commercial copper based antifouling paint (paint). Before application of the paint or nanorod growth, fishing nets were cleaned and degreased. Briefly, the nets were sonicated in soap water for 15 min and then thoroughly rinsed with deionized water until soap residues were removed. Following drying in air, the nets were sonicated in ethanol for 15 min then dried and stored in a desiccator until further use.

For nanocoating, Sol-Gel synthesis of ZnO nanoparticles was carried out by following a previously described method^58^. In brief, 4 mM sodium hydroxide was dissolved in absolute ethanol and subsequently added to 4 mM zinc acetate solution (in absolute ethanol) under continuous stirring. The mixture was hydrolysed at 60 °C for 2 hrs to form ZnO nanoparticles having a diameter of ~ 4–7 nm. The nylon nets were treated with 1% dodecanethiol solution in ethanol followed by heating at 100 °C for 15 min. Nylon net substrates were subsequently dipped into the ZnO nanoparticle colloid for 30 min, and then dried in an oven at 95 °C for 30 min. The above steps were repeated 5–7 times to obtain a proper coverage of nanoparticle seeds on the net surface. ZnO nanorods were hydrothermally grown from the seeded net substrates in a 10 mM equimolar precursor solution of zinc nitrate hexahydrate and hexamethylenetetramine. The chemical bath containing seeded net substrates and precursor solution was kept in an oven at 90 °C for 10 hrs and replenished every 5 hrs^59^. Prior to the hydrothermal process, sodium hydroxide solution was added to adjust the initial pH of the growth solution to 6.8^60^. After the preparation of the coating, the coated substrates were thoroughly rinsed with deionized water (DI) and kept in an oven at 90 °C overnight for drying. Commercial biocidal paint, Hempanet, 7177A, specifically promoted for aquaculture use was utilized in the study. This paint contain 25–50% cuprous oxide and 5% zinc oxide^20^. The paint was applied onto cleaned net strips (10 × 100 cm) by a standard paint brush representative of typically followed methods for anti-fouling paint application on aquaculture nets. After application of paint, all the substrates were dried for 2 days at ambient temperature prior to deployment in the marina.

### Surface characterization

Thermal stability of the supporting fishing net was studied using Thermo gravimetric analysis (TGA) (PerkinElmer Frontier 1, USA) (Supplementary Fig. 1). Surface morphologies of the control (unmodified), ZnO nanorods coated (nanocoating) and painted (paint) fishing nets were characterized by JEOL JSM-6301F field emission scanning electron microscope (FESEM, Japan) working at 5 kV (Optical images of coated net substrates are shown in (Supplementary Fig. 4). The compositions of control, nanocoated and painted nets were studied using Raman spectroscopy (HORIBA scientific, USA). Surface area (m^2^/g) measurements were conducted using BET surface analyzer (Micromeritics ASAP 2020 Surface area and porosity analyzer). X-ray Photoelectron Spectroscopy (XPS) (Omicron Nanotechnology, Germany) with a monochromatic Al K_α_ radiation (hν = 1486.6 eV) at a working voltage of 15 kV was used for the surface characterization of the substrates. Binding energies were calibrated according to C 1s peak at 284.6 eV. Surface wetting measurements were carried out with Theta Lite attention tensiometer (Biolin Scientific, Sweden). Water contact angle was measured five times on a dry substrate using a sessile drop of water (volume = 2 μl) and the average values are reported.

### Field experiment

Three types of samples (size: 10 cm × 100 cm) were used. Fishing net substrates were either coated with ZnO nanorods (nanocoating) or antifouling paint (paint). Fishing net substrates of the same size without any coating were maintained as control substrates. All 3 types of net substrates were attached to polyethylene pipes using polystyrene cable ties. Differently treated substrates were positioned randomly. All set-ups were deployed horizontally at a depth of 10 cm from the surface of the water body. Nets were submerged for a period of one month in the Al Mouj marina (Muscat, Oman 23°37′17″N 58°17′13″E) in January 2015. Seven replicates of each studied substrates and the control were randomly selected and collected during each sampling. Samplings were performed each week at the same collection time. At the time of sampling, sea water characteristics like temperature, pH and salinity were measured (Supplementary Table 3). The salinity of the seawater was measured using an optical refractometer (Lumen, China). Temperature measurements were carried out regularly using iButton (DS1920, USA) temperature loggers. Light intensity was measured using ISO-TECH ISM 910 (Taiwan) at the time of sample collection. pH was measured in the laboratory with Meter Toledo Seven Compact pH meter using water samples collected at the site. Fishing net substrates were collected into separate sterile plastic zip lock bags and immediately transferred to the laboratory. Biofilms were scraped from all the substrates using a sterile scalpel. Sterile seawater was used to wash out the remaining fouling from the substrates. Out of seven collected samples for each experiment, biofilms from three substrates were randomly selected and used for the estimation of total bacterial abundance. The remaining three samples were used for microbial community analysis (see below). Remaining one sample was used for scanning electron microscopy (SEM).

### Estimation of total bacterial abundance

Bacterial abundance on the replicated ZnO nanocoated, antifouling paint applied substrates and control substrates were estimated using flow cytometry (FC). FC measurements were performed using BD FACSAria^TM^ III (BD Biosciences, USA). Before FC, all the biofilm samples were filtered using cell strainer (mesh size 40 µm) to remove larger particles. Subsequently, all the samples were stained with SYBR Green I (excitation/emission wavelengths: 497 nm/520 nm; dilution 1:10000). Three FC readings were recorded for each sample. The readings were recalculated to average numbers of cells/mL.

### DNA extraction and analysis of fouling microbial communities

Part of the scraped samples were frozen and stored at −80 °C until the analysis of microbial community composition using next generation sequencing was done. DNA from each sample was extracted using a Power Biofilm MoBio Kit following the manufacturer’s instructions. Purified DNAs were analysed at Molecular Research (MRDNA, Shallowater, TX, USA) for Illumina MiSeq sequencing of the 16S and 18S rRNA genes using the primers 515F (5′-GTGCCAGCMGCCGCGGTAA-3′) and 806R (5′-GGACTACHVGGGTWTCTAAT-3′) for prokaryotes and Euk7F (5′-AACCTGGTTGATCCTGCCAGT-3′) and Euk570R (5′-GCTATTGGAGCTGGAATTAC-3′) with barcode on the forward primer^61^. Barcodes were removed and sequences with low quality and <150 bp reads were eliminated. Sequences were then denoised and chimeric sequences were detected and removed^62^. The obtained sequences were clustered at 3% divergence into operational taxonomic units (OTUs). Final OTUs were taxonomically classified using BLASTn against a database of high quality sequences derived from NCBI and RDPII. Since there were no significant differences between three replicates, the data for each treatment were pooled together and average values were used for further analysis. The OTU richness was determined by Chao1, Shannon, and Simpson diversity indices using the PAST (Oslo, USA) software.

### Scanning electron microscopy (SEM) for surface coverage

Electron microscopy was carried out using a JEOL JSM-6301F field emission scanning electron microscope (FESEM, Japan) working at 5 KV. For SEM analysis, air dried net substrates were cut into small pieces (size 5 mm × 5 mm) and were dehydrated by dipping in a series of increasing ethanol concentrations (30%, 50%, 70%, 90% and 100%). The substrates were kept in each solution for about 15 min and finally dried in a desiccator. All samples were then sputtered with platinum metal prior to loading into the microscope to avoid charging during imaging.

### Measurement of Hydroxyl radicals generated upon Photocatalysis

The formation of hydroxyl radicals (**·**OH) on the surface of net substrates was detected with photoluminescence (PL) using terephthalic acid as a fluorescence probe molecule. Terephthalic acid immediately reacts with **·**OH to produce highly fluorescent product, 2-hydroxyterephthalic acid^21^. To quantify the production of hydroxyl radicals on the surface of nanocoating, photocatalysis was carried out by irradiating 0.5 mM of Terapthalic acid in contact with each type of the substrate using continuous visible light irradiation from Solar stimulator (~AM1.5 irradiation ~1,060 W/m^2^) for a duration of about 1 hr. Light intensity measurement was performed over the surface of the sample with light intensity meter (ISO-TECH ISM 410, Taiwan) and it was observed to be 1000 W/m^2^.The PL spectrum of 2-hydroxyterephthalic acid was measured in Perkin- Elmer fluorescence spectrometer (LS 55, Santa Clara, USA). After visible-light irradiation for 1 hr, the reaction solution was collected and used to measure the intensity of the emission peak at 425 nm upon excitation by 315 nm source wavelength.

### Statistical Analysis

Using Statistica 11 (Statsoft, USA) the assumption of normality of the data were verified using the Shapiro-Wilk’s W test^63^. Factorial ANOVA was used to test the effect of nanocoatings, paint and period of immersion on the total bacterial density and production of hydroxyl radicals on the surface of the fishing net samples. Fisher LSD post hoc test was used to test significance of differences between microbial abundances on all the samples. In all cases, p value < 0.05 was considered statistically significant. Single linkage cluster analysis was used to group all samples in terms of the relative abundances (%) of bacterial and eukaryotic genera using PRIMER (Plymouth, UK) software. The effect of nanocoatings and biocidal paint on the composition of the microbial community was determined using similarity percentage (SIMPER) analyses (PRIMER, Plymouth, UK). These analyses were based on the Bray-Curtis similarity index^64^.

### Data availability

Data generated or analysed during this study are included in this article (and its Supplementary Information files). Additional datasets generated during and/or analysed during the current study are available from the corresponding author on reasonable request.

## Electronic supplementary material

Supporting information
