# Supplementary material for: Bioinspired nanocoatings for biofouling prevention by photocatalytic redox reactions
Source: Sci Rep. 2017 Jun 15;7:3624. doi: 10.1038/s41598-017-03636-6 (PMC5472575; doi:10.1038/s41598-017-03636-6)
Supplement: Supplementary file 1 — Supporting information [file 41598_2017_3636_MOESM1_ESM.pdf]

Supplementary Information

Bioinspired nanocoatings for biofouling prevention by photocatalytic redox reactions

Priyanka Sathe [a, b], Karthik Laxman [c], Myo Tay Zar Myint [d], Sergey Dobretsov [a,e*], Jutta Richter [f], Joydeep Dutta [c*]

[a] Department of Marine Science & Fisheries, College of Agricultural & Marine Sciences, Sultan Qaboos University, P.O. Box 34 Al Khoud 123, Sultanate of Oman

[b] Chair in Nanotechnology, Water Research Center, Sultan Qaboos University, P.O. Box 17, Al Khoud 123, Sultanate of Oman

[c] Functional Materials Division, Department of Applied Physics, School of Engineering Sciences, KTH Royal Institute of Technology, Isafjordsgatan 22, SE-164 40 Kista Stockholm, Sweden

[d] Department of Physics, College of Science, Sultan Qaboos University, PO Box 36, Al Khoudh, Muscat 123, Sultanate of Oman

[e] Center of Excellence in Marine Biotechnology, Sultan Qaboos University, P.O. Box 50Al Khoud 123, Sultanate of Oman

[f] Institute for Chemistry and Biology of the Marine Environment (ICBM), University of Oldenburg, Ammerländer Heerstraße 114, 26129 Oldenburg, Germany

[*]**Corresponding authors**:

1. Dr. Sergey Dobretsov: Tel.: (+968) 24143657; Fax: (968) 24413418

   E-mail: sergey@squ.edu.om

2. Prof. Joydeep Dutta: Tel. :(+46) 737652186
   E-mail: joydeep@kth.se

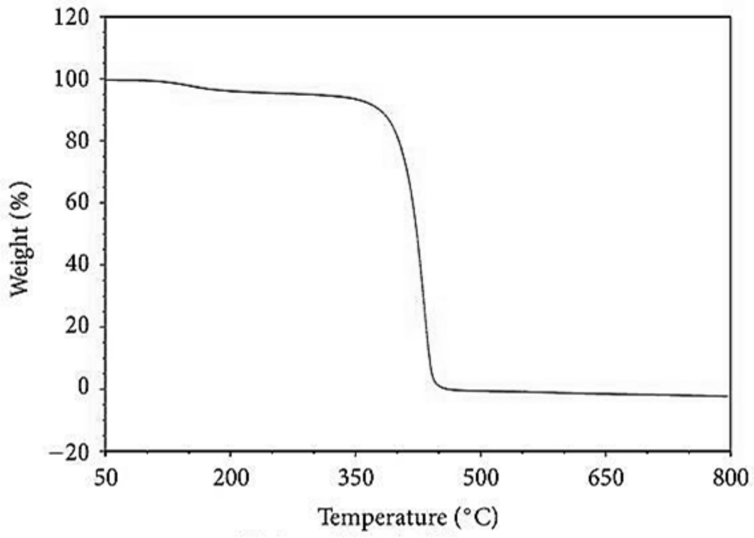

Figure 1: Shows TGA analysis of fishing net. Sample weight reduction with increasing temperature is plotted. Net substrate showed that its highly stable till the temperature reached 395ºC (it is due to the property of thermoplastic), after which it showed evaporation of the material as seen by sharp decrease in weight reduction.

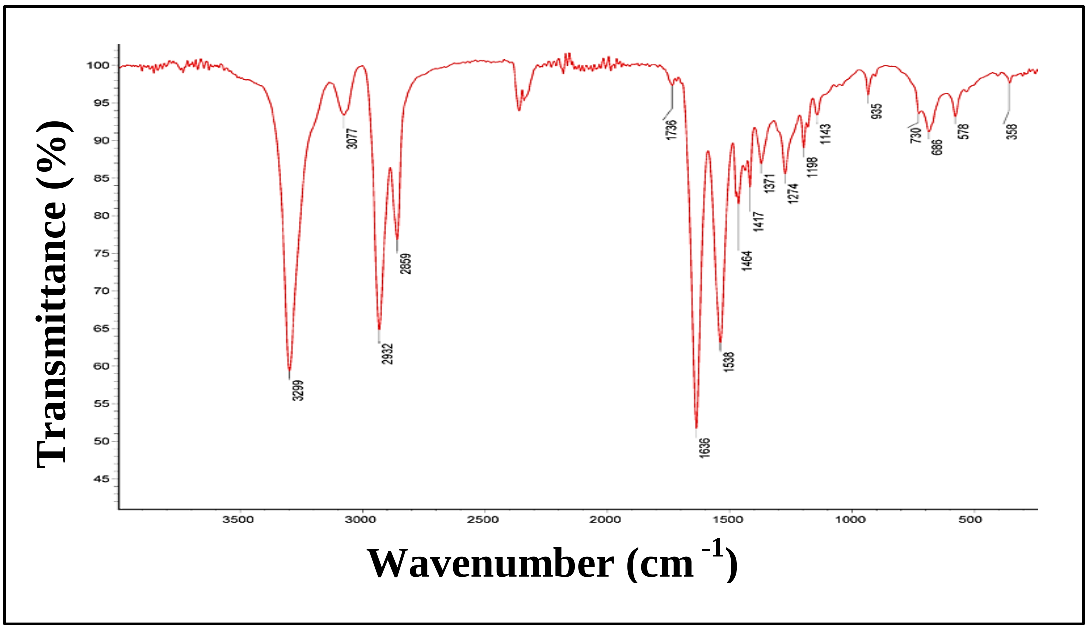

Figure 2: Typical FTIR spectrum of fishing net material. It proves that net material is Nylon 6.

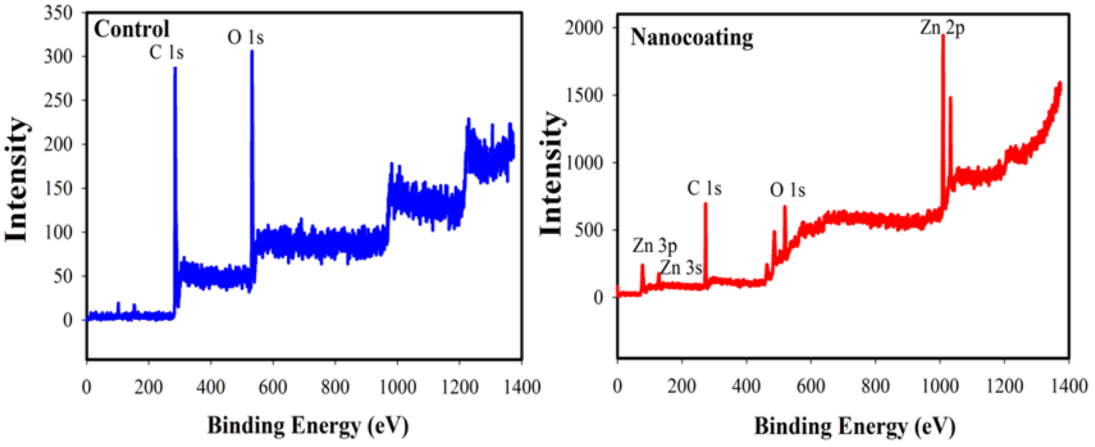

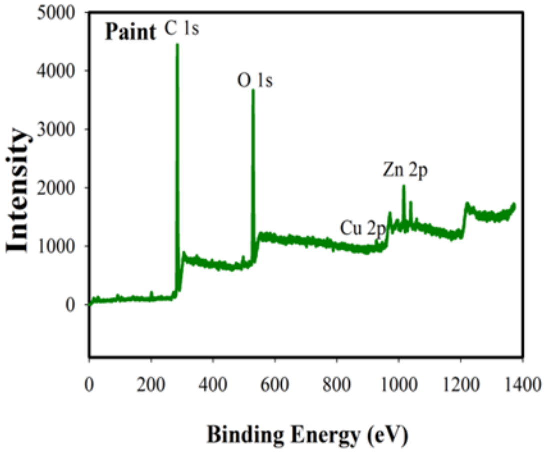

	Zn	Cu
**Paint**	11129	10515
**Nanocoating**	11491	-

Figure 3 XPS spectrum of Control, Nanocoated and painted net substrate. Adjacent table indicates area under the curve from XPS spectra for zinc and copper on the nanocoated and painted substrate.

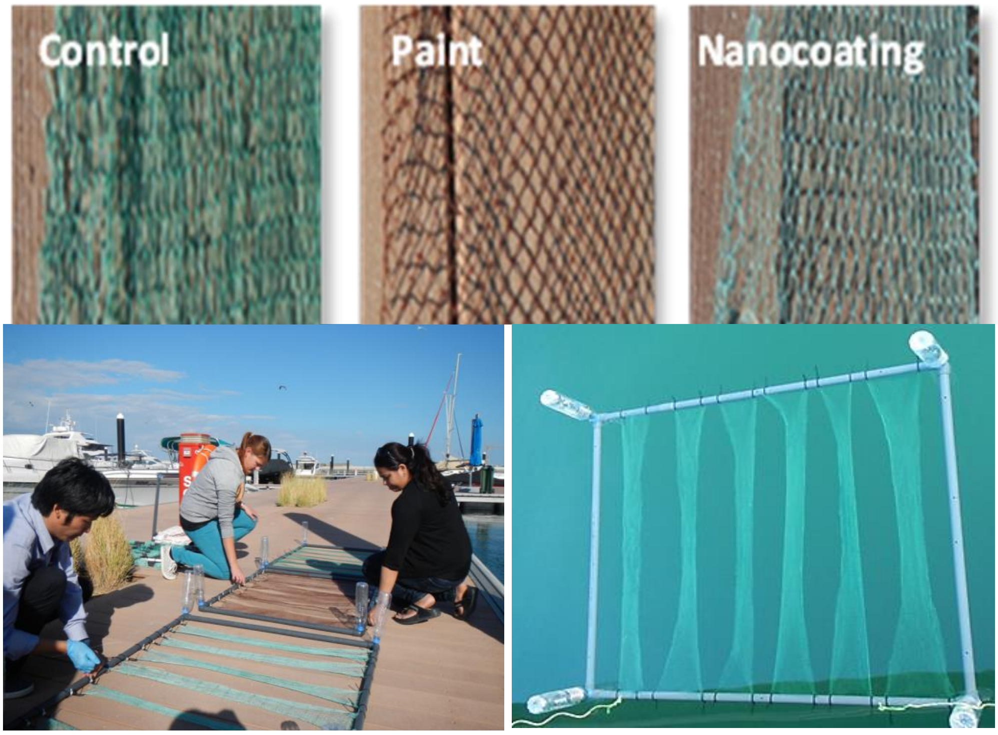

Figure 4: Photographs of all the three types of net substrate modifications used. Photographs from left shows plain nylon net, net support painted with antifouling paint and net coated with zinc oxide nanorods. Netting deployment at marina is shown in the other photographs.

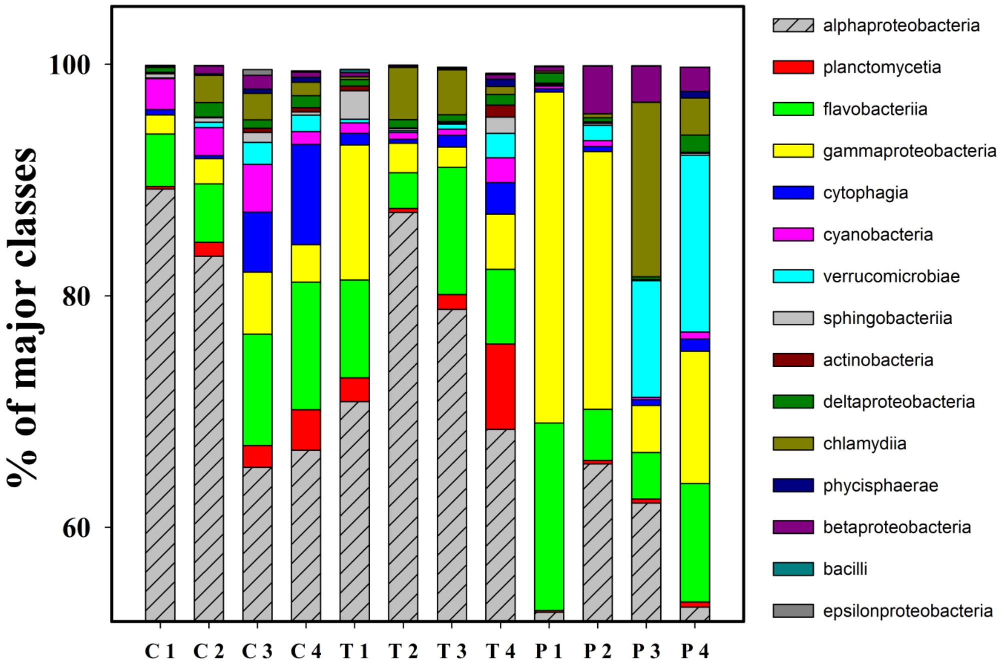

Figure 5: Stacked bar chart showing the relative abundance (%) of major bacterial classes present on the control (C), nanocoated (T) and painted (P) net substrates during exposure in

marina. C 1 to C 4- control week 1 to week 4; T 1 to T 4- Nanocoating week 1 to week 4; P 1 to P 4- Paint week 1 to week 4. Reported values represent average value obtained from three replicates. Bacterial genera contributing < 5% are not shown.

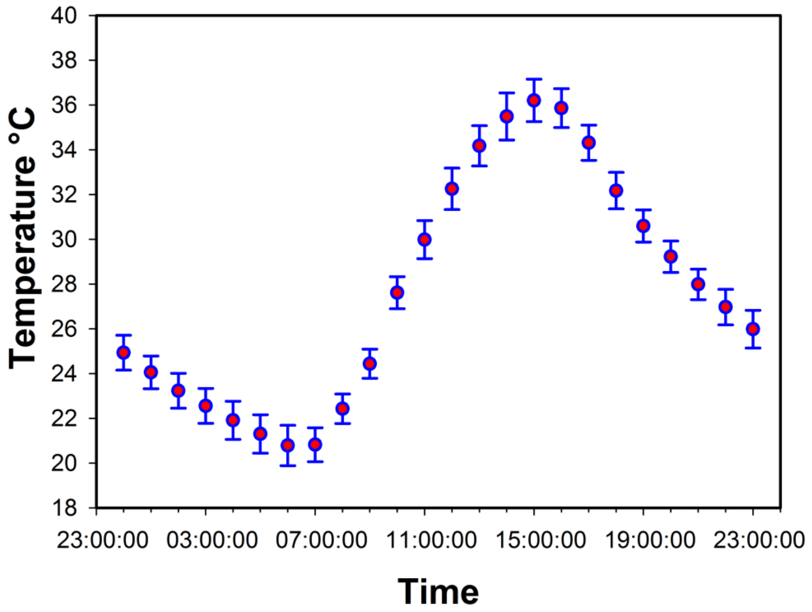

Figure 6: Temperature profiles of seawater during experimental period of 28 days. Values reported here are average temperature readings (°C) for 28 days ± standard deviation.

Table 1: Estimation of bacterial diversity indices for microbial communities developed on tested net substrates. Calculated diversity indices are based on MiSeq Illumina sequencing of the microbial communities developed on the control (C) nanocoating (T) and painted (P) net substrates over 28 days. Samples were collected weekly C1 to C4- control week 1 to week 4; T1 to T4- Nanocoating week 1 to week 4; P1 to P4- Paint week 1 to week 4

**Sample**	**Treatment**	**Collection day**	**No. of OTU's**	**Shannon**	**Chao I**	**Simpson**
C1	Control	7th day	722	2.176	11	0.222
C2	Control	14th day	750	2.173	11	0.216
C3	Control	21st day	715	2.172	11	0.223
C4	Control	28th day	425	2.172	11	0.251
T1	Nanocoating	7th day	860	2.175	11	0.290

T2	Nanocoating	14th day	389	2.176	10	0.332
T3	Nanocoating	21st day	654	1.956	10	0.262
T4	Nanocoating	28th day	225	2.172	11	0.224
P1	Paint	7th day	758	2.175	11	0.406
P2	Paint	14th day	820	1.958	11	0.473
P3	Paint	21st day	895	1.957	10	0.432
P4	Paint	28th day	630	1.956	10	0.502

Table 2: BET surface area for all the substrates

**Sample (net)**	**BET surface area (m^2/g)**
Control	0.075
Nanocoating	1.468
Paint	0.082

Table 3: Sea water quality of the test site

**Chemical characteristics**	**Week 1**	**Week 2**	**Week 3**	**Week 4**
Salinity (ppt)	37	38	38	38
pH	8.4	8.3	8.4	8.4
Light intensity over the surface (Min-Max W/m^2)	0-1100	0-1150	0-1170	0-1120
